# Serum Bcl-2 Levels in Patients with β-Thalassemia Minor: A Pilot Study

**DOI:** 10.4274/tjh.2013.0152

**Published:** 2014-12-05

**Authors:** İrfan Yavaşoğlu, Gökhan Sargın, Gürhan Kadıköylü, Aslıhan Karul, Zahit Bolaman

**Affiliations:** 1 Adnan Menderes University Faculty of Medicine, Division of Hematology, Aydın, Turkey; 2 Adnan Menderes University Faculty of Medicine, Department of Internal Medicine, Aydın, Turkey; 3 Adnan Menderes University Faculty of Medicine, Department of Biochemistry, Aydın, Turkey

**Keywords:** β-Thalassemia minor, Bcl-2, apoptosis

## Abstract

**Objective:** Anti-apoptotic proteins such as Bcl-2 and Bcl-xL may play a role in the survival of erythroid progenitor cells. Information about these proteins in patients with β-thalassemia minor is limited. We aimed to determine the levels of serum Bcl-2 in patients with β-thalassemia minor.

**Materials and Methods:** Ninety-seven patients (60 females and 37 males with mean age of 29±21 years) with β-thalassemia minor were enrolled in this study. The diagnosis of β-thalassemia minor was based on whole blood counts, family history, and HbA2 levels estimated by high-performance liquid chromatography. The control group comprised 23 healthy adults (17 females and 6 males with mean age of 58±9 years) without anemia. The levels of serum Bcl-2 were measured by enzyme-linked immunosorbent assay. Mann-Whitney U tests were used in statistical evaluation and p<0.05 was accepted as statistically significant.

**Results:** Although there was no statistically significant difference between patients with β-thalassemia minor and the control group for the level of serum Bcl-2 (p>0.05), these levels were higher in β-thalassemia minor patients than controls.

**Conclusion:** There are damaged beta chains in β-thalassemia minor. Therefore, it is expected that premature death of red blood cells may occur due to apoptosis. The mean age of the control group was higher than that of the β-thalassemia minor group; this may be why Bcl-2 levels were higher in the β-thalassemia minor group. It is known that older age constitutes a risk for increased apoptosis. Other proteins (Bad, Bax, etc.) and pathways [CD95 (Fas) ligand] associated with apoptosis should be evaluated in future studies including more patients.

## Abstract (tr)

**Amaç:** Bcl-2 ve Bcl-xL gibi antiapoptotik proteinler eritroid progenitör hücrelerin yaşam süresinde rol oynayabilir. β-talasemi minör hastalarda bu proteinler ile ilgili bilgiler sınırlıdır. Biz, β-talasemi minörlü hastalarda serum Bcl-2 düzeylerinin belirlenmesi amaçladık.

**Gereç ve Yöntemler:** β-talasemi minör tanılı doksan yedi hasta (60 kadın ve 37 erkek, yaş ortalamaları 29±21 yıl) bu çalışmaya dahil edildi. β-talasemi minör tanısı tam kan sayımı, aile öyküsü ve yüksek performanslı sıvı kromatografisine dayanan HbA2 düzeyleri ile konulmuştur. Kontrol grubu anemisi olmayan 23 sağlıklı yetişkin (17 kadın ve 6 erkek, yaş ortalaması 58±9 yıl) idi. Serum Bcl-2 düzeyleri ELISA yöntemi ile ölçüldü. İstatistiksel değerlendirmede Mann-Whitney U testi kullanıldı ve p<0.05 istatistiksel olarak anlamlı kabul edildi.

**Bulgular:** Serum Bcl-2 düzeyinde β-talasemi minörlü hastalar ile kontrol grubu arasında istatistiksel olarak anlamlı farklılık olmamasına rağmen (p>0.05), β-talasemi minörlü hastalarda bu seviyeler kontrol grubuna göre daha yüksekti.

**Sonuç:** β-talasemi minörde hasarlı beta zincirleri bulunmaktadır. Bu nedenle, kırmızı kan hücrelerinin erken ölümünün apoptoz nedeniyle olması beklenmektedir. Kontrol grubunun yaş ortalaması talasemi taşıyıcılarından yüksektir, bundan dolayı Bcl-2 düzeyleri β-talasemi minörde yüksek olabilir. İleri yaşın artmış apoptoz için bir risk oluşturtuğu bilinmektedir. Bu nedenle, 40 yaş üzerindeki talasemi minörlülerde, kontrole göre Apoptoz ile ilişkili diğer protein (Bad, Bax, vb.) ve yolaklar [CD95 (Fas) ligand] hasta sayısının daha fazla olduğu çalışmalarda değerlendirilmelidir.

## INTRODUCTION

Translations or mutations of mRNA lead to a deficiency of globin synthesis and thus cause thalassemia syndromes [1]. Thalassemia syndromes are inherited disorders that occur as a result of abnormal synthesis of α/β-globin. Cytoplasmic inclusion bodies (including unpaired globin molecules) damage red blood cells. As a result, the life span of erythrocytes is shortened [1,2].

Cell death is regulated by many intra- and extracellular signals. The ratio of anti-apoptotic molecules (Bcl-xl, Mcl-1, Bcl-w, A1, etc.) to apoptotic molecules (Bax, Bak, Bik, Bid, etc.) determines the future of the cell [3]. The Bcl-2 proto-oncogene is located on chromosome 18 and inhibits the apoptotic pathway. Bcl-2 is an important molecule in the early period of cell transformation [3,4].

In recent years, some studies have been published about Bcl-2 genes to predict their role in the development of many solid tumors. However, there are few studies about the expression of Bcl-2 in patients with β-thalassemia minor [5].

In this study, we aimed to determine the levels of serum Bcl-2 in patients with β-thalassemia minor.

## MATERIALS AND METHODS

Ninety-seven patients (60 females and 37 males with mean age of 29±21 years) with β-thalassemia minor were enrolled in this study. The diagnosis of β-thalassemia minor was based on whole blood counts, family history, and HbA2 levels estimated by high-performance liquid chromatography (Agilent 1100 Series HPLC Value System, Waldbronn, Germany). The control group comprised 23 healthy adults (17 females and 6 males with mean age of 58±9 years) without anemia. The levels of serum Bcl-2 were measured using a commercial enzyme-linked immunosorbent assay kit (Biosource, Cat. No. TMA 0311, Camarillo, CA, USA). Age, sex, hemoglobin, hematocrit, mean corpuscular volume (MCV), whole blood cell counts, and serum levels of Bcl-2 were recorded. Venous blood samples were taken under the supervision of medical personnel and were measured with an ADVIA 2120 instrument (Siemens, Erlangen, Germany). Signed informed consent was obtained from all participants. SPSS 15 (SPSS Inc., Chicago, IL, USA) and the Mann-Whitney U test were used in statistical evaluation of data and p<0.05 was accepted as statistically significant.

## RESULTS

**Figure 1:**
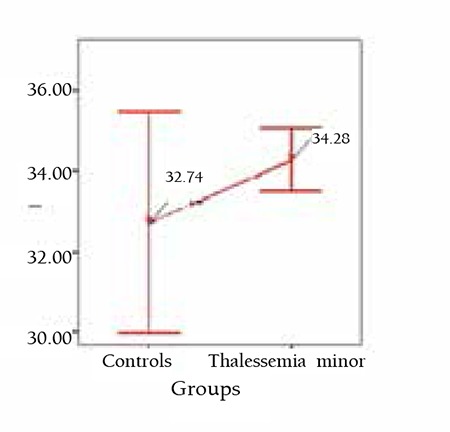
Serum Bcl-2 levels with dot-plotdistribution.

In Table 1, age, hemoglobin, MCV, and serum Bcl-2 levels are given. Although there was no statistically significant difference between patients with β-thalassemia minor and the control group for the level of serum Bcl-2 (p>0.05), levels in β-thalassemia minor patients were higher than in the controls. Bcl-2 levels of the patients with β-thalassemia minor and the control group are shown by dot-plot distribution in Figure 1. The relationships between age, hemoglobin, and MCV values and serum Bcl-2 levels are shown in Table 2.

We evaluated serum Bcl-2 levels of 22 controls and 27 patients with β-thalassemia minor of over 40 years. Bcl-2 levels were statistically significantly higher among patients than in the control group (p=0.045).

## DISCUSSION

In this study, we did not find any significant difference in Bcl-2 levels between patients with β-thalassemia minor and controls.

Antiapoptotic proteins (Bcl-2, Bcl-xL, and Mcl-1) are necessary for the survival of erythroid precursor cells [6]. Proteins such as Bax and Bcl-xS trigger apoptosis of erythroid cells. According to some studies, Bcl-2 and Bax genes may be regulated by the p53 gene. If the ratio of anti-apoptotic proteins to pro-apoptotic proteins is more than 1, erythroid cells continue their life span [7]. Additionally, members of the Bcl-2 family may control apoptosis by acting as pro-oxidant agents [8].

Erythropoietin increases Bcl-xL expression in erythroid-CFU colonies [5]. Significantly increased Bcl-xL and Mcl-1 protein levels and the suppression of Bax protein are observed in CD34 (+) cells, induced by erythropoietin or stem cell factor [9]. Myelodysplastic syndrome (MDS) is characterized with ineffective erythropoiesis and increased apoptosis of hematopoietic precursor cells. A correlation was observed with increased proteins associated with Bcl-2, but the mechanism is not yet fully understood [10].

Levels of Bad, Bax, and Bcl-xS were especially higher in patients with MDS-refractory anemia (RA) or RA with ring sideroblasts [10]. Many changes in oncogenes and tumor suppressor genes may have a role in the pathogenesis of many diseases. Bcl-2 is located in the nucleus or membranes of the endoplasmic reticulum, while mitochondria were shown to be in neoplastic cells in B-cell neoplasms with t(14;18). The mechanism of Bcl-2 activation is still unknown; however, increased levels of cytochrome-c as a center of apoptosis may play a role [11]. Hockenbery et al. reported that Bcl-2 inhibits apoptosis by changing mitochondrial functions [12].

Translations or mutations of mRNA lead to deficiency of globin synthesis and thus cause thalassemia syndromes. It was reported in many studies that some cancers may be treated by regulation genes on pre-RNA and mRNA regions. Changes in apoptotic or anti-apoptotic pathways may be a result of genetic modulation [13].

β-Thalassemia is characterized by accelerated apoptosis of erythroid precursor cells. In many studies, limited data were reported about these proteins and genetic mechanisms in patients with thalassemia [2,3,4,5]. Increased synthesis of fetal hemoglobin may improve the symptoms of β-thalassemia. Cytotoxic drugs such as hydroxyurea and cytarabine may affect the synthesis of fetal hemoglobin by stimulating precursor cells [1]. Castaneda et al. determined that short-chain fatty acids [arginine butyrate, sodium α-methylhydrocinnamate, sodium 2,2-dimethylbutyrate, sodium 3,4-(methylenedioxy) cinnamate, 2-(quinazolin-4-ylamino) butanoic acid, and 4-(trifluoromethyl) sulfanyl aniline acetic acid] increased the erythroid-BFU colonies and endogenous fetal globin gene expressions [5]. In addition, anti-apoptotic genes were specifically regulated and Bcl-2 levels were increased by short-chain fatty acids. It is known that increased apoptosis is observed with older age [14].

Cao et al. reported that neutrophils of patients with paroxysmal nocturnal hemoglobinuria expressed apoptosis-related CD95, Bcl-2, and Bax without significant differences from the normal controls [15]. The results of a study by Ismail et al. suggest a more significant role for Bcl-x as an anti-apoptotic regulator in CD34 (+) cells in aplastic anemia than Bcl-2 [16].

The limitations of our study were the existence of an age gap between the 2 groups and a limited number of cases. Moreover, we could not evaluate other anti-apoptotic proteins (Bad, Bax, etc.) or pathways [CD 95 (Fas) ligand] in this study. The control group comprised a small number of patients and the control group comprised 23 healthy adults without anemia. Another limitation of this study is that we did not know the levels of annexin V, cleaved caspase-3, or mRNA regulation of Bcl-2.

In conclusion, there are damaged beta chains in β-thalassemia minor. Therefore, it is expected that premature death of red blood cells may be due to apoptosis. The mean age of the control group was higher than that of the β-thalassemia minor patients, which may be the reason for Bcl-2 levels being higher in the β-thalassemia minor patients. It is known that older age constitutes a risk for increased apoptosis. Other proteins and pathways associated with apoptosis should be evaluated in future studies including larger populations.

**Conflict of Interest Statement**

The authors of this paper have no conflicts of interest, including specific financial interests, relationships, and/or affiliations relevant to the subject matter or materials included.

## Figures and Tables

**Table 1:**
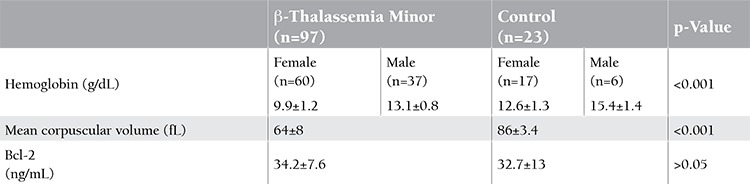
Hemoglobin, mean corpuscular volume, and serum Bcl-2 levels in patients with β-thalassemia minor and the control group.

**Table 2:**
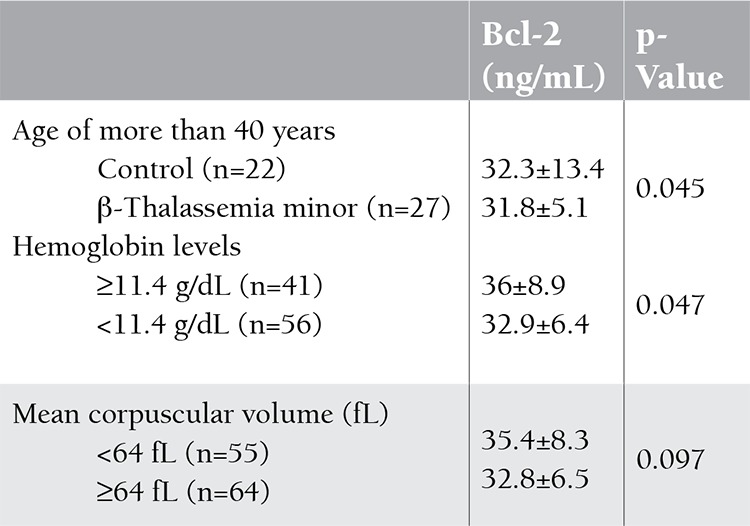
The comparison of serum Bcl-2 levels between age and mean corpuscular volume groups.
